# Standing Genetic Variation and the Evolution of Drug Resistance in HIV

**DOI:** 10.1371/journal.pcbi.1002527

**Published:** 2012-06-07

**Authors:** Pleuni Simone Pennings

**Affiliations:** Harvard University, Department of Organismic and Evolutionary Biology, Cambridge, Massachusetts, United States of America; Utrecht University, Netherlands

## Abstract

Drug resistance remains a major problem for the treatment of HIV. Resistance can occur due to mutations that were present before treatment starts or due to mutations that occur during treatment. The relative importance of these two sources is unknown. Resistance can also be transmitted between patients, but this process is not considered in the current study. We study three different situations in which HIV drug resistance may evolve: starting triple-drug therapy, treatment with a single dose of nevirapine and interruption of treatment. For each of these three cases good data are available from literature, which allows us to estimate the probability that resistance evolves from standing genetic variation. Depending on the treatment we find probabilities of the evolution of drug resistance due to standing genetic variation between 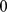 and 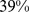. For patients who start triple-drug combination therapy, we find that drug resistance evolves from standing genetic variation in approximately 6% of the patients. We use a population-dynamic and population-genetic model to understand the observations and to estimate important evolutionary parameters under the assumption that treatment failure is caused by the fixation of a single drug resistance mutation. We find that both the effective population size of the virus before treatment, and the fitness of the resistant mutant during treatment, are key-parameters which determine the probability that resistance evolves from standing genetic variation. Importantly, clinical data indicate that both of these parameters can be manipulated by the kind of treatment that is used.

## Author Summary

For HIV patients who are treated with antiretroviral drugs, treatment usually works well. However, the virus can, and sometimes does, become resistant against one or more drugs. HIV drug resistance results from the acquisition of specific and well known mutations. It is currently unknown whether drug resistance mutations usually stem from standing genetic variation, i.e., they were already present at low frequency before treatment started, or whether they tend to occur during treatment. In the current manuscript, I make use of several large datasets and evolutionary modeling to estimate the probability that drug resistance mutations are present before treatment starts and lead to viral failure. I find that for the most common type of treatment with a combination of three drugs, drug resistance evolves from pre-existing mutations in 6% of the patients. With other types of treatment, this probability varies from 0 to 39%. I conclude that there is room for improvement in preventing the evolution of drug resistance from pre-existing mutations.

## Introduction

For most HIV patients, treatment with modern antiretroviral therapy leads to a rapid decline of viral load (VL) of several orders of magnitude. However, when the virus acquires resistance to one or more drugs, treatment can fail. It is still an open question whether the mutations responsible for resistance originate usually from standing genetic variation (also referred to as pre-existing mutations or minority variants), or from new mutations which occur during therapy. In fact, there is no single biological system for which the relative role of pre-existing and new mutations is well known [1]. Another important open question is whether multiple simultaneous mutations are needed for the viral population to be able to grow during therapy, or whether a single mutation allows escape. Amongst evolutionary biologists, it is commonly assumed that therapy with multiple drugs works so well because the virus needs multiple mutations to escape, which is unlikely to happen. However, patient data show that patients often fail therapy with a single resistance mutation [2], [3] which suggests that a single mutation can increase the fitness of the virus to above 1, even though the virus is still susceptible to two of the drugs in the treatment. In this scenario, the main benefit of combination therapy over monotherapy would be that combination therapy reduces the population size of the virus and therefore the probability that mutations occur. In this study we will analyze patient data under the assumption that a single mutation can lead to virologic failure and thereby propose an alternative view on the evolution of drug resistance during multi-drug therapy.

We will look at the establishment of drug resistance mutations in three different situations: (1) when triple-drug therapy (ART) is started for the first time, (2) when pregnant women are treated with a single dose of nevirapine (sdNVP) to prevent infection of the baby during birth and (3) when ART is interrupted and restarted (an overview of abbreviations is given in table 1). We will argue that standing genetic variation plays a crucial role in each of these cases. We find that the probability that resistance mutations become established in each of these cases can be understood by using a simple population genetic model.

**Table 1:** Abbreviations.

Abbreviation	Explanation
VL	Viral load, the number of viral particles per ml blood
ART	Antiretroviral therapy, here used to mean treatment with two NRTIs and an NNRTI or an “unboosted” PI
PMTCT	Prevention of mother to child transmission
DRM	drug resistance mutation
NRTI	Drug of class nucleoside reverse transcriptase inhibitor
NNRTI	Drug of class non-nucleoside reverse transcriptase inhibitor
PI	Drug of class protease inhibitor, PIs can be used “unboosted” or “boosted” with an additional drug.
NVP	Nevirapine, an NNRTI
sdNVP	Single dose nevirapine
ZDV	Zidovudine, also known as AZT, an NRTI
3TC, DDI, FTC, TDF	Drugs of NRTI class
PP	Post partem, used here for drugs which are added to sdNVP right after the mother has given birth

For readers who are not familiar with HIV, it is important to know that the genotype-phenotype map for drug resistance in HIV is very well known. Lists of the important resistance mutations for each drug are published (e.g., in the International AIDS Society–USA drug resistance mutations list, [4], so that doctors can compare the genotype of the virus of a patient before treatment with this list to decide which drugs to prescribe. The aim of treatment is to achieve viral suppression. If treatment fails, i.e., the viral load stays or becomes higher than a predetermined threshold, such as 50/ml, despite adherence to the regimen, a second genotypic test will be performed to see whether the virus has acquired new resistance mutations. Since the second half of the 1990s, treatment is usually with a combination of three drugs, which are chosen such that mutations which confer resistance against one of the drugs do not confer cross-resistance against the other two drugs. Soon after its introduction, it became clear that triple-drug therapy was an enormous success and saved the lives of many HIV patients [5]. One reason why therapy with three drugs works better than treatment with one or two drugs is that the rate at which resistance evolves is slower when patients are treated with three drugs [6]. It is commonly thought that resistance does not evolve in patients on triple-drug therapy because it would require a viral particle to acquire three mutations at the same time. However, in patients who are treated with triple-drug therapy, it is often observed that resistance against one of the drugs evolves, at least initially. Data from several cohort studies in different parts of the world, such as from Canada [2] and the UK (UK CHIC cohort study [3], clearly show that in most patients who fail therapy due to resistance, the virus is resistant against one of the drugs and almost never against all three. The UK study, for example, reports that out of 4306 patients who started therapy between 1996 and 2003, after two years of therapy, 13% have drug resistance. A majority of the patients with drug resistance (7%) have resistance against just one of the drugs. Less than half of the patients (6%) have resistance against more than one class of drugs and a only small number of patients (1%) have resistance against 3 classes of drugs, even though all patients of this cohort were treated with three classes of drugs. These data show that treatment can fail due to resistance against one of the drugs in a regimen. In such cases, it may be that the other two drugs cannot keep the VL completely suppressed, even though they still work. The viruses that have acquired resistance against two or three classes of drugs may have acquired these mutations at the same time or they may have acquired them one by one. For now, we will assume the latter and focus only on the probability of acquiring the first drug resistance mutation (DRM).

For many common drugs, especially reverse transcriptase inhibitors, a single mutation can confer resistance against the drug and only a small number of mutations is responsible for resistance in most patients. For example, resistance against the drug nevirapine is almost always due to one of two amino acid changes, namely K103N or Y181C in the reverse-transcriptase gene [7]. Because of the importance of a small number of mutations, several studies have investigated whether these mutations are present in untreated patients due to transmitted drug resistance or due to spontaneous mutation. Recent studies have used allele-specific PCR and related methods to determine the frequency of several important mutations in untreated patients. Low-frequency drug resistance mutations (DRMs), likely due to spontaneous mutation (and not transmitted from other patients) were detected in up to 40% of patients (see [8] for an overview). The detection of drug resistance mutations in untreated patients, together with the knowledge that a single mutation can confer resistance against a drug and allow viral escape, suggest that pre-existing resistance mutations (or standing genetic variation in the population genetic jargon) may play an important role in the evolution of drug resistance in HIV.

Throughout the paper, we will assume that a single mutation can allow viral escape and we focus on the probability that such a first drug resistance mutation becomes established (i.e., it reaches such a frequency that it can be expected to become the majority variant unless treatment is stopped or changed quickly). What happens after a first mutation has become established, or how fast such an established mutation wanes in the absence of treatment are important questions, but they fall outside the scope of this study. In this paper, “triple-drug therapy” and ART refer to treatment with two drugs of the class NRTI plus either an NNRTI or an unboosted PI (for a list of abbreviations in the paper, see Table 2). The results are likely to be different for other drug combinations.

**Table 2:** Parameter values for analytical predictions and computer simulations.

Parameter	Value	Explanation
**Values roughly based on literature**		
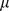	5*10−5	Mutation rate to resistant genotype
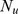	2000	Effective population size in untreated patient
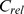	0.05	Relative cost of mutant in absence of therapy
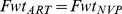	0.5	Absolute fitness of wildtype during therapy
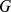	200	Number of HIV generations per year
**Values estimated in current study**		
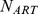	108	Effective population size in patient on ART
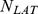	5	Number of activated latent cells in patient on ART
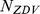	1000	Effective population size in patient on ZDV monotherapy
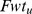	1.62	Absolute fitness of wildtype in absence of therapy (determines growth rate during treatment interruption)
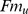	1.54	Absolute fitness of resistant mutant in absence of therapy
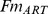	1.017	Absolute fitness of resistant mutant during ART
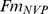	1.54	Absolute fitness of resistant mutant during NVP therapy
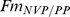	1.025	Absolute fitness of resistant mutant during NVP/PP therapy

### Starting therapy

When a patient starts therapy for the first time, one would expect that there should be a substantial probability that drug resistance evolves due to pre-existing DRMs. Indeed, recent studies have shown that the presence of drug resistance mutations at low frequency (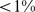) increases the risk that treatment fails (e.g., [9],[10], [7], see [11] for a review). However, the situation is not as simple as one may hope: even if no pre-existing DRMs can be detected, resistance mutations may become established quickly, and even if DRMs are detected, treatment is still successful in the majority of patients. We will attempt to understand those observations using population genetic theory. Other authors have looked at the question of pre-existing DRMs previously (e.g., [12], [13]), however, it is worth reconsidering the topic. First of all, we now have a wealth of data available for pre-existing DRMs and the establishment of drug resistance mutations in HIV patients, and secondly, we now have a better theoretical framework to consider the role of standing genetic variation for adaptation [14].

### Prevention of mother to child transmission (PMTCT)

Pregnant women in low resource settings are often treated with a single dose of the non-nucleoside reverse transcriptase inhibitor neverapine when labor starts. Single dose nevirapine (sdNVP) is the cheapest and simplest way to reduce the probability of mother-to-child-transmission, but it is shown to lead to the establishment of drug resistance mutations in the mothers and the babies. In a meta-analysis [15], found that, in 7 different studies, on average 44% of the patients treated with sdNVP had detectable NVP resistance mutation several weeks after the treatment. The presence of such mutations makes future treatment of these women harder [16]. To avoid the establishment of resistance mutations, several alternative strategies are used in combination with sdNVP. We will use the same population genetic framework as in the other two cases to try to understand why sdNVP leads to establishment of resistance mutations in so many patients, and how this can be avoided. In the current study we will only focus on the probability that NVP resistance mutations become established during treatment for PMTCT. The issue of how these mutations wane and possibly resurface when treatment is started again is important and interesting but falls outside the scope of the current paper.

### Treatment interruptions

It was long suspected that treatment interruptions lead to drug resistance. Indeed, cohort studies show that treatment interruptions due to non-adherence are associated with faster accumulation of drug resistance mutations ([17], [18], [19]. Clear evidence that treatment interruptions of at least a couple of weeks lead to the establishment of resistance mutations comes from clinical trials (e.g., [20], [21], [22] which were done in a time when it was believed that treatment interruptions may be beneficial for patients. In 2006 the SMART trial was stopped because treatment interruptions were shown to have a negative effect on patients' health [23]. However, treatment interruptions still occur, for example, when a patient is forgetful or is unable to purchase drugs due to financial or logistic barriers. It is important to understand how treatment interruptions lead to resistance and whether this effect can be avoided.

The main idea that currently governs the thinking about treatment interruptions and resistance is that insufficient drug-levels allow for replication and, at the same time, select for resistance (e.g., [24], [25], [18]. This effect is aggravated when drugs that are part of combination therapy have very different half-lifes, so that interrupting combination therapy can result in effective monotherapy. It is generally believed that this “tail of monotherapy” is the main reason why treatment interruptions lead to drug resistance. However, several observations are not compatible with the “tail” hypothesis. For example, Fox et al ([25]) found no significant difference in the number of resistance mutations after simultaneous, “staggered” or “switched” treatment interruptions in patients from the SMART trial (a “staggered” stop means that the long half-life drug is interrupted several days before the other drugs and a “switched” stop means that before interrupting, patients switch to a regimen with only short half-life drugs). In addition, the “tail” hypothesis fails to explain why treatment interruptions increase the risk of resistance in patients on protease inhibitor-based (PI) regimens which do not have long half-lifes [26], [20], [27], [28], [29], [30]. Another explanation is therefore needed to understand the observed patterns.

When treatment is interrupted, the viral load rapidly increases until it has reached its original level after approximately four weeks [31]. Basic population genetics tells us that such population growth also leads to an increase in the probability that DRMs are present. When treatment is started again, selection may work on such pre-existing mutations, which provides a simple explanation for how treatment interruptions lead to the establishment of resistance mutations.

In this paper we will attempt to explain the observed patterns by considering selection on pre-existing variation and selection on new mutations. It is worth noting here that pre-existing does not necessarily mean old, such a mutation may have originated just a day before the start of treatment. Throughout the paper, we use a mathematical model for adaptation from standing genetic variation which we developed previously [14] and forward-in-time, individual-based computer simulations. The model captures mutation, drift and selection, including changing selection pressures (due to stopping and starting of therapy) which lead to changes in population size. Because we only focus on the establishment of the first drug resistance mutation, we can ignore epistatic interactions between different drug-resistance mutations and recombination. In each of the three cases of interest, we use published data on the percentage of patients with established drug resistance mutations to estimate important parameter values (for starting ART or sdNVP) and to predict outcomes (for treatment interruptions).

## Model

### Model, assumptions and fixation probability of a drug resistance mutation

The model we use in this paper describes the population dynamics and population genetics of a panmictic viral population in a single patient. Details of the model can be found in the supplementary material. We assume that as long as the patient is not treated, the viral population will be stable at population size 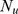 (u for untreated). Drugs reduce the fitness of the wildtype virus to below 1 so that the population will shrink. We assume that there is a large reservoir of latently infected cells of which a fixed number (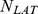) become activated per generation, so that the virus can not die out. Drug resistant virus can be created by mutation and is assumed to be resistant against one of the drugs in the treatment regimen. If the patient is not taking drugs, the drug resistant virus is less fit than the wildtype by a factor 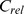 (relative cost of the resistant virus), but if the patient is taking drugs, the resistant virus has a fitness that is higher than 1 (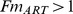), whereas the wildtype has a fitness lower than 1 (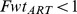). In reality, there may also be resistance mutations that confer resistance against one of the drugs, but that do not lead to a fitness higher than 1. Such mutations will quickly die out and can safely be ignored in the model. Throughout the paper we focus on the the processes that allow a first major drug resistance mutation to become established in the patient. Patients are assumed to be ART-naive and have no transmitted drug resistance.

Evolutionary biologists have long known that most mutations will be lost by genetic drift even if they confer a fitness benefit [32]. This is also true for drug resistance mutations (DRMs) in patients on anti-retroviral therapy, although it is all too often ignored in drug resistance studies. The clinical relevance of this old result has recently become very clear. It was found in several studies that even though low frequency DRMs increase the risk of treatment failure and establishment of drug resistance, the majority of patients with detected low frequency DRMs will respond well to treatment [7]. This result shows that DRMs can die out, even if they have reached frequencies high enough to be detected. The reason is probably that most viral particles will not infect any new cells and produce no new viral particles, even if, on average, they produce more than 1.

The probability that a DRM becomes established in the patient depends on the number of copies that are present, the average number of offspring of the drug resistant particles and the variance in offspring number. Traditionally, fixation or establishment probabilities are calculated using the relative fitness difference between the mutant and the wildtype, but in the case of HIV it is more useful to use the fitness of the mutant virus to calculate its establishment probability. The reason is that anti-retroviral therapy works so well that wildtype fitness may be very low (much lower than 1). In such case fitness of the mutant may not be related to the fitness of the wildtype and because the wildtype cannot grow, the two types do not compete for resources. In other words, the mutant can occupy a niche that is not occupied by the wildtype. In those cases, and as long as 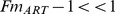, the establishment probability of the mutant will be approximately 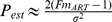 where 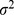 is the variance in offspring number. In the simulations and throughout this paper, we use the variance effective population size, in which case one can assume that 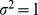, so that
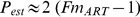
(1)


Note that by setting 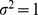, we ignore all mutations which occur in virus which is not part of the effective population size. The establishment probability of a mutation in a random viral particle (e.g., when observed in a patient) may be much lower. It is important to realize that if the establishment probability of a DRM depends on its absolute fitness, anything that reduces its fitness will reduce the establishment probability. For example, if a drug that is added to a regime reduces fitness of both wildtype and resistant virus, then it will reduce the probability that a pre-existing resistant mutant becomes established. This is true even if the effect of the added drug on wildtype and resistant virus is exactly the same. Similarly, if the immune system works well, this may also reduce the probability of establishment.

In most population genetics models, the focus is on the fixation probability, rather than the establishment probability of a mutation. And in many models, if a mutation becomes established, it will go to fixation. However, if selection pressures change, establishment does not necessarily lead to fixation. This is especially clear when we will later consider the effect of a single-dose of nevirapine. A few weeks after a single dose of nevirapine, nevirapine resistance mutations can be detected in a large proportion of patients, but these mutations may never take over the whole viral population, because the treatment duration is very short and wildtype virus will quickly become a majority again (see for example, [16]). In fact, the standard results on fixation probability [32] are really results on establishment probabilities, so we can use them without problems.

### Psgv vs. Pnew

For drug resistance to evolve, the viral population needs viral particles that carry drug resistance mutations. Such particles may already be present before treatment is started. To denote this possibility we use 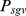 or the probability that drug resistance establishes from the standing genetic variation. If the mutation is not already present, or if was present but was subsequently lost, then the viral population has to wait for a new mutation to occur and become established. We denote this possibility as 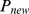, or the probability that resistance evolves due to new mutations. In the latter case, we have to indicate a time window, such as per year or per generation.

The goal of this study is to understand and, albeit roughly, quantify 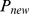 and 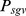 for HIV drug resistance in patients on triple-drug regimes (consisting of an NNRTI or an unboosted PI plus two NRTI's) and in patients who are treated with single dose nevirapine.

## Results

### Starting standard therapy

When a patient starts anti-retroviral therapy for the first time, the viral population in that patient will move from an equilibrium without drugs to an equilibrium with drugs. At the pre-treatment equilibrium, the viral population size will at its equilibrium level (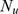), and resistance mutations are expected to be at mutation-selection-drift equilibrium, where most mutations will be present at very low frequencies (see, e.g., [7]). Note that mutation-selection-drift equilibrium is reached quickly for mutations that are very costly to the virus. So even though it may take years for neutral diversity to reach an equilibrium level in an HIV patient [33], important drug resistance mutations which are 5 or 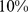 less fit than the wildtype are expected to reach their (dynamic) equilibrium in weeks or months.

Standard population genetic theory predicts that the average frequency of a resistance mutation is equal to the mutation rate (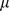, per viral particle and per replication) divided by the relative cost (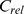) of the resistance mutation, though drift causes actual frequencies to vary greatly between different time points and between patients (see also [34]). Even though the average frequency is independent of the population size, in larger populations, it is more likely that DRMs are present and the absolute number of drug resistant particles will, naturally, be higher. When treatment starts, resistance mutations will confer a fitness benefit to the virus and they can (but are not guaranteed to) increase in frequency and become established. The probability that this happens depends on the number of resistant particles in the population and on the establishment probability of a mutation that is present in a single particle. In [14] we derived formulas to calculate the probability that adaptation to a new environment happens from the standing genetic variation (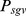). We will use the approximate equation 8 in [14]:
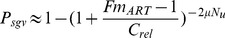
(2)


It is also possible to use the the number of resistant particles in a patient (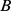), if this is known, and the fitness of these copies (in the environment with drugs) to calculate the probability that a resistance mutation becomes established:
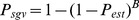
(3)where we use the probability that all copies of the resistance mutation die out to calculate the probability that at least one survives. The probability that resistance mutations become established increases with the number of copies of resistant virus and the probability that any one of these survives.

### Evolution of resistance during therapy

If resistance did not evolve from standing genetic variation, it may evolve due to new mutations. The probability that this happens in a given year will depend on the number of generations (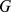) in a year, the mutation rate (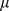), the effective population size during antiretroviral treatment (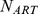) and the establishment probability of a mutation (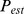). In principle, the establishment probability during therapy may not be the same as in the very beginning of therapy, for example because the number of available cells which a particle can infect could be different. However, throughout this paper we will assume that 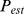 depends only on the kind of therapy and not on how long a patient has been treated. Using a poisson approximation, we find that the per year probability that resistance evolves is
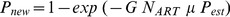
(4)


It is debated whether during therapy, there is ongoing replication or whether a reservoir of latently infected cells is entirely responsible to residual viremia. If the reservoir reflects the composition of the viral population before treatment, then the expected frequency of the resistance mutation in the reservoir would be 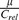. If the number of latently infected cells that become activated every generation is 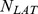, then the expected number of activated cells with resistant virus would be 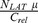. The per year probability that resistance evolves due to activated cells from the reservoir would be
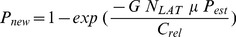
(5)


It is also possible that there is ongoing replication, but that the reservoir also plays a role at the same time, so that the reality will be reflected best by a combination of equations 4 and 5. Note that 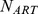 and 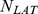 are both effective population sizes, and may be much lower than the census population sizes.

### Comparison with data and parameter estimation

Published data show that the rate of evolution of drug resistance is roughly constant over long times (see for example the study by [3], in which patients were followed for up to eight years). This fits with expectations if 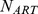 and 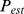 remain constant so that 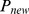 stays constant. However, several studies show that the probability that resistance mutations become established is higher in the first year of therapy, as compared to later years. This can be seen, for example, in a study on a large cohort in British Columbia, especially when one considers the most adherent group of patients (figure 2 in [35], see also [17]). A similar effect is seen in [11] when one considers the patients with pre-existing DRMs. This effect, that resistance is more likely to evolve in the first year of therapy as compared to later years, can be easily explained by standing genetic variation.

Under the assumption that 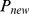 is indeed constant, we can use published data to estimate both 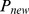 and 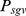. Margot et al [36] reported the number of patients in which resistance was detected in the first, second and third year after treatment initiation in a cohort of patients who were treated with NNRTI-based ART. The reported data (see table S1 in supplementary text S2) show that the probability that resistance was detected in the first year was 9.5%, whereas in the second and third year it was only 3.7% (see supplementary material for details on how this was estimated). The difference of 5.8% is likely due to standing genetic variation at the start of therapy.

We will use the estimates for 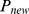 (0.037 per year) and 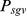 (0.058) from [36], in combination with other, published, estimates to get a rough estimate of the important evolutionary parameters. First of all, we will assume that the mutation rate from one nucleotide to a specific other nucleotide is 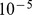 [37], so that if there are five main resistance mutations for a given drug combination, the total mutation rate is approximately 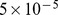. For the remainder of the paper, we will only use this total mutation rate. If the mutation rate would be higher (lower) than our assumption, the estimated population sizes would be lower (higher) than our estimates. An overview of the parameter values we use in the paper is given in table 2.

We know that the important drug resistance mutations are at least somewhat costly for the virus. Their cost, 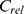, has been estimated for several drug resistance mutations, both in vivo and in vitro (for an overview on resistance mutations in the reverse transcriptase gene see [38]). For example, [39] find that the relative cost of resistance mutation M184V is approximately 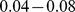. Wang et al [40] estimate a cost of 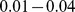 for K103N, which is the most common NNRTI resistance mutation. Other studies were not able to detect any cost of K103N, but given its low frequency in untreated patients [7], it seems likely that it is associated with a significant cost. In this paper we will use an average cost of 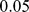 for all mutations.

**Figure 1:**
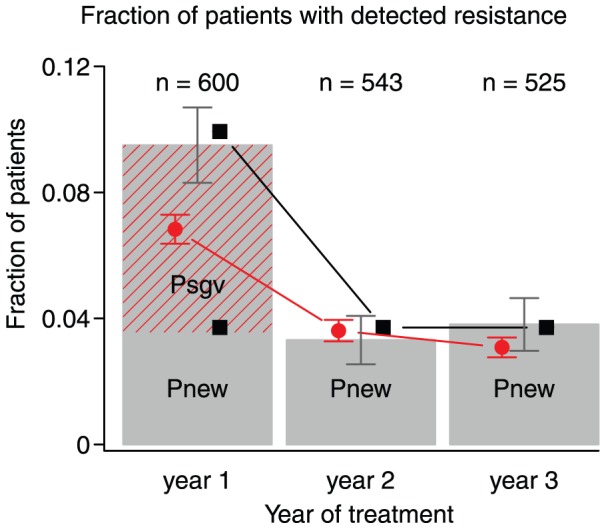
Probability of detecting resistance per year of treatment. The probability that resistance is detected for the first time in the first, second or third year of treatment, given that it was not detected until then. Grey bars are the estimates from the Margot et al ([36]) dataset, and the number of patients on which the estimates are based are noted at the top of the graph. The red dashed area reflects the inferred probability that resistance mutations from standing genetic variation become established. The black squares are values calculated using equations 2 and 4. The red circles are estimated from 1000 simulations. Parameters as in table 2.

Given the cost, the mutation rate, 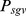 and 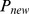, and using the assumption that there are 200 HIV generations in a year [41], we can find the combinations of 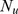, 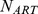 and 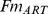 that are compatible with the data (shown in figure 1). Estimates for the effective population size in untreated patients range from 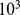 [42] to 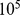 [43]. We know that a large proportion of untreated patients carries low frequency drug resistance mutations, but not all patients, which gives us some additional information about the population size in an untreated patient (see figure 1b). If we choose a value of 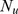 of 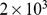, then we find that about half of the patients should carry pre-existing DRMs. This is somewhat higher than what is usually detected, but that can be due in part to the limits of detection of current tests [8]. An overview of the parameter estimates that were used in the simulations and for analytical predictions can be found in table 2.

Given our choice of 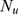, we find that 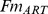 must be approximately 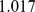, leading to 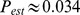. Under the assumption 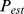 stays the same during treatment, the Margot et al data are compatible with a 18-fold reduction of the effective population size due to therapy, to an effective population size of 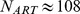 . Note however, that the estimate of a 18-fold reduction depends heavily on the assumption that 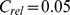 . For example, had we assumed a 10% cost, then the estimated reduction would have been 37-fold , and for a 1% cost, the reduction would have been only 4-fold . The reason is that if we assume that costs are high, then we must also assume that the mutant fitness (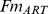) is relatively high, in order to find 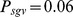, and if 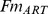 is high, 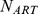 must be low, to explain 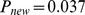.

If the evolution of resistance during therapy is not due to ongoing replication, but due to continuous activation of latent cells, then, under the assumption that 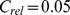, the effective number of cells (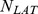) must be approximately 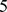 per generation. This means a reduction of effective population size of almost 400-fold. However, it is not so clear whether in this case the word “population size” should still be used, because the number 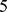 is not an estimate of the size of the reservoir, but an estimate of the effective size of the part of the reservoir that is reactivated every generation.

The result that the frequency of resistance mutations in the reservoir depends on their fitness cost (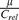), whereas the cost does not play a role for new mutations due to ongoing replication, could be harnessed to estimate the relative importance of the reservoir. If the reservoir is the most important source of resistance mutations during therapy, then the same set of mutations should be found in patients whose virus acquires resistance quickly after the start of therapy and in those who acquire mutations during therapy. However, if ongoing replication is the source of resistance mutations during therapy, then mutations with a high cost in the absence of drugs should occur relatively more often during therapy than quickly after therapy is started.

**Figure 2:**
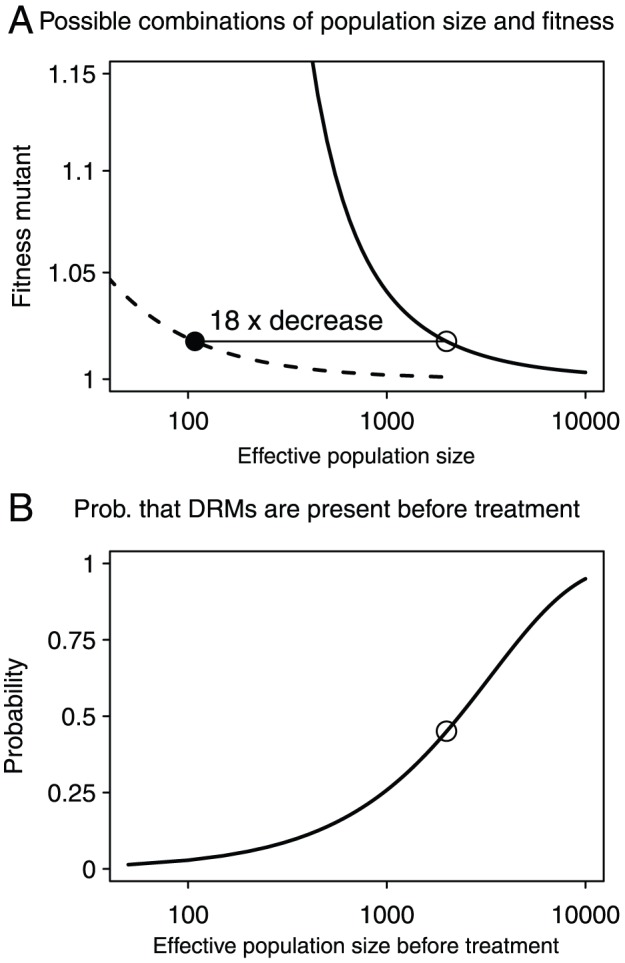
Possible combinations of population size and fitness and the effect of population sizes on the probability that DRMs are present before treatment. **Figure 2a:** Continuous line: combinations of population size before treatment (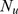) and fitness of mutant virus during therapy (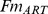) that lead to the observed probability that resistance mutations from standing genetic variation become established (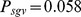). Dashed line: combinations of population size during treatment (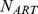) and fitness of mutant virus during therapy (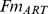) that lead to the observed probability that resistance mutations from standing genetic variation become established (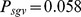). Open dot: 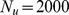 and 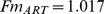, closed dot: 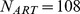, 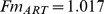. **Figure 2b**: Probability that a patient has any pre-existing DRMs before the start of therapy for different population sizes, and 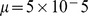. Open dot: 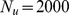.

The data and the results from simulations and predictions (using equations 2 and 4) are shown in figure 2. The percentage of patients with resistance after one year is lower in the simulations than in the analytical predictions, because in the simulations, it takes time for a mutation to increase in frequency and be detected. We assume that it is detected as soon as it is more frequent than the wildtype, the result is that in the simulations (and probably in reality) 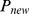 is lower in the first year than in the other two years. It is unclear how large this effect is in reality, but it means that the 6% we find is a conservative estimate of the role of standing genetic variation. If it would take 3 months for a mutation to increase in frequency and become detected, then 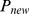 in year 1 would be 75% of its value in the later years, and 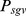 would be approximately 7% in stead of 6%.

### Single-dose nevirapine for prevention of mother-to-child-transmission

A single dose of nevirapine (sdNVP) just before labor starts reduces the risk that a mother transmits HIV to her baby at birth, but leads to high levels of resistance in many women. Because of the long half life of nevirapine, even a single dose lasts at least a few days. However, this is a very short amount of time (only a few HIV generations) so that probably most or all detected NVP resistance mutations are due to standing genetic variation.

Because it is known that sdNVP can lead to the establishment of resistance mutations, and also to further reduce the risk that the baby becomes infected with HIV, several different treatment strategies are being used. In this study, we focus only on those strategies that include a single dose of nevirapine (and exclude, for example, pregnancy limited triple-drug therapy). Basically, sdNVP can be combined with either a short course of zidovudine monotherapy during the third trimester of pregnancy (ZDV/sdNVP), or it can be combined with additional drugs during and after labor up to one month postpartum (sdNVP/PP). It can also be used alone (sdNVP) or combined with both (ZDV/sdNVP/PP), resulting in four possible strategies.

Under the assumption that all resistance is due to standing genetic variation, it is straightforward to predict, at least qualitatively, the effect of the four treatment options. Single dose nevirapine plus two additional drugs (sdNVP/PP) is a three drug regimen, and similar to standard antiretroviral therapy (ART), except that it only lasts a few days or weeks. We therefore expect similar levels of drug resistance due to standing genetic variation. If only NVP resistance is considered (and not resistance to the other two drugs), we expect to find somewhat lower levels than in the normal case, although the difference may not be large because resistance against NVP is more common than resistance to most other drugs. Treating with only sdNVP is different from starting ART, in that there is only one drug. The result is that the fitness of both wildtype and resistant virus will not be reduced as much as in the normal case. Specifically, NVP resistant virus will have a relatively high fitness during NVP monotherapy. This high fitness (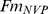) leads to a high establishment probability (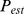) for available resistance mutations. In fact, the establishment probability may be so high that in virtually all patients that carry some NVP resistance before treatment, the resistant virus will increase in frequency during NVP treatment.

An interesting treatment option is to start with a few weeks of ZDV monotherapy before treating with a single dose of nevirapine. The ZDV treatment will reduce the population size of the virus, 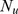, so that the probability that NVP resistance is available and the copy number of such resistant mutants if they are available will be lower by the time the patient is treated with NVP. ZDV monotherapy ultimately leads to ZDV resistance, but the risk that resistance mutations become established during a short course is small. ZDV monotherapy reduces the viral load approximately three-fold [44]. Finally, adding ZDV treatment before labor and two additional drugs during and after labor (ZDV/sdNVP/PP) will reduce both the availability of NVP resistant virus and the establishment probability of such virus, which should lead to an even lower probability that NVP resistance mutations from standing genetic variation become established.

### Comparison with data for single dose nevirapine

**Figure 3:**
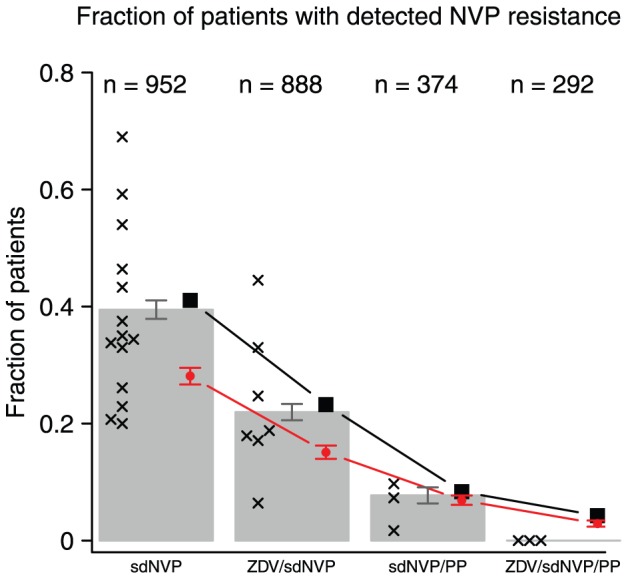
The probability that resistance mutations are detected in women treated for prevention of mother-to-child transmission. The probability that resistance mutations are detected 6 to 8 weeks after treatment with single dose nevirapine. Black crosses are data from single studies, grey bars with estimated standard error are percentages for all studies combined (the number of patients that were used to calculate this percentage is indicated at the top of the graph). Red circles with standard error are results from 1000 simulations and the black squares are analytical predictions. Parameter values as in table 2.

We identified 23 published studies that reported on NVP resistance 6 to 8 weeks after women were treated with sdNVP. Several of the studies directly compared two different treatment options. We found at least three studies for each of the four different treatment options. An overview of the studies can be found in table S2 in the supplementary text S2. For each study we recorded which of the four treatment options was used and in how many of the patients NVP resistance mutations were detected using simple Sanger (population) sequencing (we excluded studies that only recorded deep-sequencing or allele-specific PCR results, as there were too few of those to allow us to compare the treatment options). For each of the four treatment options, we also calculated the overall probability that resistance mutations were detected in a patient (simply by summing the number of patients with resistance and summing the total number of patients in the studies). We found that sdNVP leads to detectable resistance mutations in 39% of 952 patients, ZDV/sdNVP leads to detectable resistance mutations in 22% of 888 patients, adding two drugs during and after labor (sdNVP/PP) lead to detectable resistance mutations in 7.8% of 372 patients and ZDV/sdNVP/PP lead to detectable resistance mutations in none of 292 patients (see figure 3).

**Figure 4:**
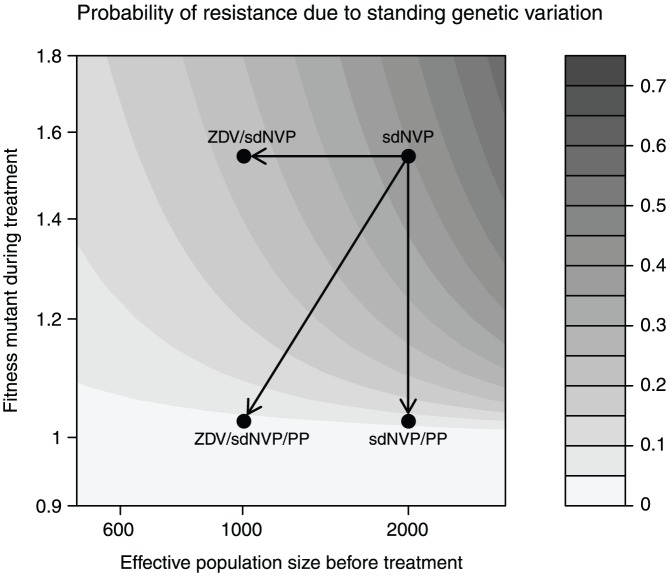
Probability of the establishment of DRMs as a function of effective population size and the fitness of the resistant mutant during treatment. The predicted probability of the establishment of drug resistance mutations from standing genetic variation depending on the effective population size and the fitness of the resistant mutant during therapy. Grey scales indicate the probability of the evolution of drug resistance due to standing genetic variation. Dots indicate estimated parameter combinations for treatment with just sdNVP, with ZDV monotherapy followed by sdNVP (ZDV/sdNVP), with sdNVP followed by two additional drugs postpartum (sdNVP/PP) and with ZDV monotherapy followed by sdNVP and two additional drugs postpartum ZDV/sdNVP/PP.

We now used these data, in combination with our previous parameter estimates, to estimate the fitness of a NVP resistant mutant during NVP therapy (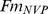) and the reduction of the population size due to ZDV treatment (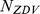). We find that 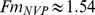 and that ZDV reduces the effective population size approximately two-fold (table 2 and figure 4). The results show that a reduction in population size by ZDV monotherapy does reduce the probability that NVP resistance mutations become established, but adding two drugs to sdNVP helps much more. We also estimate the fitness of the mutant during therapy with nevirapine and two additional drugs and find a slightly higher value than our previous estimate (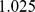 vs 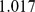), though these differences are not statistically significant.

### Interruption of therapy

**Figure 5:**
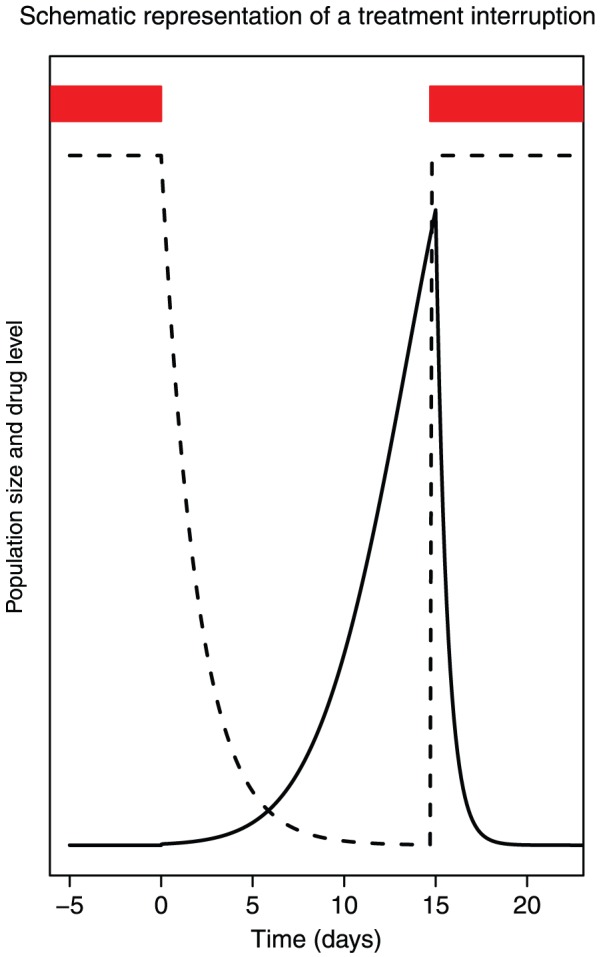
Drug level and population size during and after a treatment interruption. Drug level (dashed line) and viral population size (solid line) during and after a treatment interruption. Red bars indicate when drugs are taken.

During a treatment interruption, drugs are first removed from the body, which can take from a couple of hours to a several days or even weeks ([45], [46], [47]. With some delay, depending on the half-life of the drugs, the viral population begins to grow, which is observed as an increase of viral load (see figure 5). Published data show that after treatment is stopped, viral load quickly increases in almost all patients (e.g., [2]. Davey et al [31] show that average viral load plateaus four weeks after treatment is interrupted. Garcia et al [48] and Trkola et al ([49]) both report that a plateau is reached between four and eight weeks after treatment interruptions. An interruption is ended when treatment is started again and viral load goes down, hopefully to undetectable levels. Figure 1 shows a cartoon of the pharmacodynamics and population dynamics of a treatment interruption.

### Restarting therapy

If the length of a treatment interruption is so long that the population size is back to pretreatment level and mutation-selection-drift equilibrium is again reached, the probability that resistance mutations become established when therapy is started again will equal the probability that resistance mutations become established the first time a patient starts treatment, 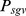 from equation 2. But if a treatment interruption is shorter than that, it is hard to calculate the exact probability that resistance will evolve upon re-initiation of therapy because neither population-dynamic, nor population-genetic equilibrium will have been reached. The absence of the population-genetic equilibrium is most problematic if resistance mutations are not very costly to the virus. However, for a costly mutation it takes only on the order of 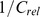 generations to reach mutation-selection-drift equilibrium. The absence of population-dynamic equilibrium is less problematic, because it is relatively easy to predict the population size of the virus or to measure viral load. In the simulations, we allow the population to grow exponentially until it reaches the baseline level. The resulting population size can be plugged into equation 2 to get an estimate of the probability that resistance mutations become established due to a treatment interruption.

### Comparison with data for treatment interruptions

**Figure 6:**
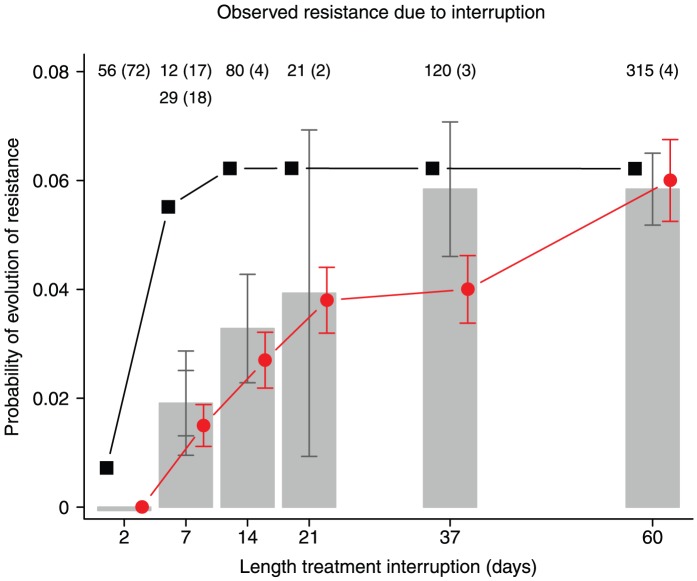
The relationship between the length of a treatment interruption and the probability that DRMs become established. Estimated probability that resistance mutations become established due to a single treatment interruption. Grey bars are data from seven clinical trials, 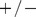 estimated standard error (see supplementary table S3 in text S2). The number of patients (and the number of interruptions per patient) are noted at the top of the graph. The red circles are estimated from 1000 simulations, 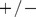 estimated standard error. The black squares are predictions using the average population size from the simulations and equation 2. Parameters as in table 2.

Using the parameter values from the last two sections, we can predict the risk that resistance mutations become established due to a treatment interruption of a certain length. We use the estimated fitness of the mutant virus during NVP therapy, and assume that the fitness of the mutant in absence of drugs is the same. With that value, we can calculate the fitness of the wildtype in the absence of drugs, because of the assumption that the cost of the resistance mutation is 5%. The wildtype fitness will determine how fast the virus grows in the simulations after treatment is interrupted, and therefore how long it takes before the population size is back at the pretreatment level. Specifically, we use 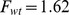. In the simulations, the population size plateaus after just 14 days, but 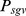 reaches its expected value only after 60 days (figure 6).

We collected information from structured treatment interruption trials to test the predictions. The probability that resistance mutations become established due to a single treatment interruption was estimated for seven clinical trials with different lengths of treatment interruptions [50], [22], [51], [52], [53], [54], [20]. An overview of the trials can be found in table S3 in text S2 (supplementary material). We first calculated the risk under the assumption that all observed resistance was due to treatment interruptions and then subtracted the estimated probability that resistance mutations become established during therapy. The corrected values are shown in figure 6. The data show that longer treatment interruptions indeed lead to a higher risk of resistance. The risk plateaus around 37 days, which is consistent with the time it takes for viral load to reach its equilibrium level (although the simulations suggest that the risk should plateau later than the population size). The highest risk was found to be approximately 6% per interruption, just like the risk of starting therapy for the first time.

## Discussion

The main aim of our study was to understand and quantify the importance of standing genetic variation for the evolution of drug resistance in HIV. We find that the probability that at least one resistance mutation becomes established due to standing genetic variation (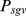) depends on the kind of treatment chosen. Most clearly, it is much higher when treatment is with sdNVP (which is monotherapy) than if treatment is with triple-drug combination therapy. For standard combination therapy (ART), we use two different data sources to estimate the probability that resistance mutations from standing genetic variation become established. In the first part of this paper we used data on the number of patients in which resistance was detected in the first year of treatment versus later years. In the third part of this paper we used data from clinical trials on treatment interruptions. In both cases, we found that the probability that resistance mutations from standing genetic variation became established was approximately 6%.

The importance of new mutations as compared to pre-existing mutations could be estimated from the Margot et al ([36]) study. We estimated that the probability that a resistance mutation becomes established during therapy (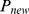) is 3.7% per year, which means that pre-existing mutations and new mutations are equally important after about one-and-a-half year of treatment. Two of the interruption studies also provided estimates for 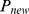, which were slightly higher (4.3% and 4.8% per year) than the estimate from the Margot et al [36] study (see table S3 in text S2). It is likely that some of the patients in these studies were not perfectly adherent to treatment, so that our estimate of 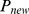 is inflated by patients who interrupted treatment. This does not affect our estimates of 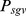. However, it means that the relative importance of pre-existing mutations is highest in completely adherent patients (because new mutations are relatively unimportant for them) and lower in non-adherent patients (see [7] but see [11]).

A stochastic model was used to understand the effect of standing genetic variation on the evolution of drug resistance during HIV treatment. Four parameters are crucial to understand the role of standing genetic variation. Three of them determine the amount of genetic variation that is available (effective population size, mutation rate and cost of the resistance mutations) and one determines how likely it is that the available mutations become established (the absolute fitness of the resistant virus during treatment).

The cost and the mutation rate are parameters that are different for each specific mutation. Together, they determine the expected frequency of the mutant in an untreated patient. For example, in untreated patients the frequency of K103N was found to be lower than the frequency of Y181C [7], suggesting that 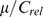 is lower for K103N. The costs for some of the most important mutations (M184V, K103N) have been estimated and are between 1 and 10% ([38], [39], [40]. Throughout this paper we used a value of 5%.

The effective population size in an untreated patient (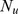) determines how much variation there is in the frequency of resistant mutants between patients. If 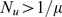, the frequency in each patient will be very close to the expectation, 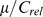, but if 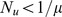, there will be a lot of variation between patients, and in many patients no resistance mutations may be available at all. Data suggest that in HIV the latter is the case (e.g., [7]), which means that, not every single point mutation is created every generation in an HIV patient. Or, more precisely, each mutation may be created, but not in a viral particle that is part of the effective population size. Mutations may even be detected in the blood stream of a patient, but may still be irrelevant if the viral particles with the mutations are eliminated before they can infect a CD4 cell. 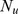 also determines the number of resistant viral particles in a patient with a given frequency of the mutant. With higher 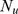, there will be a higher number of resistant particles, and this makes it more likely that resistance mutations become established when treatment is started [11].

We find that data are compatible with an 18-fold reduction of 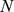 due to ART and a two-fold reduction of 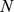 due to ZDV monotherapy. The estimated reduction depends on the assumed cost of mutations; if we assume that mutations are twice as costly, we would find a reduction that is twice as severe. Still, the reductions we find are not nearly as severe as one may have expected based on viral load reductions. During ART, VL may be reduced 1000-fold or more (in the Margot ([36]) study from which we used the data, patients had a viral load of, on average, 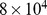 before treatment, whereas after 48 weeks of treatment, about 80% of the patients had a viral load of less than 50, [55]). This discrepancy may be due to two effects: firstly, our estimate is an average for many patients and this average may be driven up by patients in which the drugs do not work well, or who are not adherent to therapy so that their VL does not go down as much as expected. Secondly, the relationship between effective population size and viral load may not be linear, so that a thousand-fold reduction in VL may translate in only a twenty-fold reduction in effective population size.

The fourth important parameter is the fitness of the mutant virus during treatment (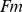), which determines the establishment probability (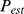).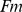 will depend on both the drugs that are used and on the specific mutation. For example, the resistance mutation K103N is more likely to become established during sdNVP than during triple-drug therapy, because additional drugs reduce 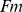 (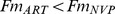). And during triple-drug therapy, K103N is more likely to become established than Y181C (even though Y181C is present at higher frequencies before treatment), likely because 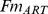 is higher for K103N than for Y181C.

### Starting of standard therapy

We assumed that the rate of evolution due to new mutations is constant and that the establishment of a resistance mutation from standing genetic variation leads to viral failure and is detected within one year of starting therapy. Maybe the most convincing evidence for these assumptions comes from the Li et al [11] study, where their figure 2 shows that (1) patients without detected pre-existing DRMs show a constant rate of evolution of resistance and (2) patients with detected pre-existing DRMs show an increased rate compared to the patients without pre-existing DRMs, but only in the first year of treatment. We used these assumptions to estimate the probability that resistance mutations from standing genetic variation become established. However, the estimated role of standing genetic variation may be a slight underestimate, because establishment of new mutations should need some time so that 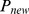 would normally be somewhat lower in the first year of treatment. The observation that the effect of standing genetic variation only lasts a year, means that fixation of a resistance mutation must take less than a year. This limits possible values for 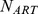 and 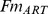 to such values for which the fixation time is less than 200 generations.

If resistance indeed evolves due to standing genetic variation in 6% of patients on standard ART, then there is clearly room for improvement. Note that those 6% of patients have already lost their first treatment option shortly after having started treatment. They have to switch to second-line treatment which is more expensive, usually more complicated (more pills per day) and likely has more side effects. It is therefore worth exploring ways to avoid the establishment of resistance mutations from standing genetic variation. Figure S2 suggests two options to reduce 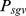, by reducing the population size or by reducing the fitness of the resistant mutants. The first may be achieved by ZDV monotherapy, as shown in the section on PMTCT, whereas the second may be achieved by adding additional drugs to the treatment. Obviously, triple-drug combination treatment is already standard for most HIV patients, but it may be worth considering specifically which treatment options would be best to prevent the evolution of resistance from standing genetic variation. This may mean, for example, to add a fourth drug to the therapy in the first couple of weeks of treatment. Resistance to boosted PI's is very uncommon, so they may be a good choice for starting treatment, in combination with two or three other drugs.

### Resistance due to sdNVP

Studying treatment with a single dose of nevirapine gives us a unique opportunity to study the effect of standing genetic variation, because treatment is so short (only a few HIV generations) that we can assume that most or all resistance mutations that are detected are from standing genetic variation. Data show that the risk that resistance mutations become established due to such treatment is very high (39%). We find that this high probability can be explained entirely by selection on pre-existing drug resistance mutations, because the fitness of NVP resistant virus is probably very high during NVP monotherapy. We estimate that its fitness is approximately 1.5. The probability that a resistance mutation becomes established can be reduced by either adding additional drugs to lower the fitness or by lowering the population size so that fewer mutants are available. A study from Zambia [56] showed that the additional drugs even help to reduce the establishment of NVP resistance mutations considerably if the additional drugs are given as a single dose (in stead of treatment for a couple of days or weeks). We did not include this study in the overview, because there was only one study that looked at this treatment option.

The results on ZDV/sdNVP/PP treatment (i.e., treatment with ZDV during pregnancy and NVP plus two other drugs during labor) are surprising in that NVP resistance mutations were not detected in any of the women who received this treatment, even though the model would predict that mutations would be detected in 4% of the women. Most of the data on this treatment option are from the Lallemant [57] paper (222 women). In this study, the authors do find some mutations that confer resistance to the NRTI's in the study (in 2.3% of the women). The same study also looked at women who were treated with ZDV/sdNVP and also in these women the percentage with resistance mutations was very low (6.4%) and much lower than the mean value for women who receive this treatment (22%). The reason for the surprisingly low values of drug resistance in this study could be that the women in the study had very low viral loads (median 2800). This probably also means that they have a low effective population size. It therefore seems unlikely that the extremely good results from the Lallemant study [57] can be replicated in other populations. However their results still show that using additional drugs to reduce the population size and to reduce the fitness of the mutant may be a good strategy to reduce the probability that resistance becomes established.

### Treatment interruptions

Considering treatment interruptions, our model provides several testable predictions. 1) resistance mutations are more likely to become established after long treatment interruptions when viral loads are higher, 2) the risk that resistance mutations become established due to a treatment interruption can not be larger than the risk at the start of treatment, 3) treatment interruptions increase the risk of establishment of resistance mutations even for drugs with short half-lifes.

Data from seven clinical trials show that indeed, longer interruptions increase the probability that resistance mutations become established (figure 6). Moreover, the estimated probability appears to plateau after 37 days, which is similar to the time it takes for viral load to reach its pretreatment level. This suggests that the risk of establishment of resistance mutations is directly linked to the viral load when treatment is started again. The second prediction was also found to hold: the estimated risk that resistance mutations from standing genetic variation become established at the start of treatment was found to be similar to the risk due to a long treatment interruption (6% in both cases). The third prediction also holds, as data show that interruptions increase the risk of establishment of resistance mutations even for PI based treatment [20], [51], where the “tail of monotherapy” cannot explain the observations.

A potential problem with the data is that not only the length of the interruptions, but also the length of treatment periods between the interruptions differed between the seven studies. The trials that were compared also differed in the drugs that were used (see table S3 in text S2), which makes direct comparison difficult. Despite all these limitations, it becomes clear that longer interruptions carry a higher risk of evolution of resistance than shorter interruptions.

If interruptions lead to the establishment of resistance mutations only due to the “tail of monotherapy”, as is usually assumed in the HIV literature [24], [25], [18], we would predict that: 4) treatment interruptions increase the risk that resistance mutations become established only for drugs with long half-lifes, 5) the risk that resistance mutations become established due to a treatment interruption is unrelated to the risk at the start of treatment and 6) the largest risk would be due to an interruption with a length that is exactly the time it takes for the last drug to lose its effect on the wildtype virus. All of these predictions do not hold. This is not to say that the “tail of monotherapy” is not important at all. But it does show that on its own, the “tail of monotherapy” cannot explain the risk that resistance mutations become established due to treatment interruptions. When one considers possible intervention strategies, this may be good news. If treatment interruptions are risky because of restarting rather than stopping therapy, this would give doctors a possibility to reduce the risk that resistance mutations become established even after a patient has already stopped taking his or her drugs. The establishment of resistance mutations at re-initiation of treatment may be avoided by pretreatment (such as with ZDV) to reduce the availability of mutations or by using more drugs or higher doses in the first weeks of treatment to reduced the establishment of pre-existing mutations.

### General remarks

We have used a population-dynamic and population-genetic model to study several patterns of drug resistance in HIV. The model explains why resistance mutations are likely to become established in the first year of standard treatment, in women who are treated with a single dose of nevirapine and in patients who interrupt treatment. In all three cases, standing genetic variation can explain the observations.

Our results illustrate that for adaptive evolution to happen, selection and the creation of new variation need not happen at the same time, if selection can work on standing genetic variation. In the case of antiretroviral treatment, this means that insufficient drug levels (which allow for replication and selection at the same time) are not a necessary condition for the evolution of drug resistance. This result about time-heterogeneous drug levels is similar to the result on heterogeneity in space by Kepler and Perelson [58], who showed that genetic variation may be created in compartments where drugs cannot penetrate whereas selection happens in other compartments.

Our model provides a simple and quantitative explanation for why resistance is less likely to evolve when patients are treated with multiple drugs in stead of just one drug. Additional drugs reduce the fitness of a mutant that is resistant against one drug, and therefore the establishment probability of such a resistant mutant. In addition, additional drugs reduce the population size of the virus and thereby the creation of new resistance mutations. This means that there will be fewer resistance mutations with lower establishment probabilities, together leading to a strong reduction in the probability that resistance evolves. In newer therapies with boosted PIs, drug resistance has become very rare [3], which may be because boosted PIs are so strong that no single mutation can lift the virus' fitness above 1.

The model in this study may be relevant to other diseases than HIV. For example, the evolution of resistance is a problem in chronic myeloid leukemia (CML) which is a cancer of white blood cells. A recent study suggested that the probability that drug resistance evolves in CML goes down with time because the population size of the cancer goes down with time [59].

Resistance is also a problem in tuberculosis (TB), and in TB it is also known that treatment interruptions increase the risk of evolution of resistance [60]. This effect may also be due to an increased population size during the interruptions. In general, stopping treatment may be risky in cases where treatment has to be started again, which is always the case for HIV and often for TB. Each time therapy is started, resistance mutations from standing genetic variation may become established, and even if this risk is only a few percent it adds up quickly when patients interrupt treatment regularly.

## Supporting Information

Text S1 Description of the model, the simulations, the calculation of the fixation probability and the data that were used for the analysis.
(PDF)

Text S2 Text S2 includes tables S1, S2, S3. **Table S1:** Number of patients with at least one resistance mutation detected by the end of the first, second and third year of NNRTI-based antiretroviral therapy. Data from [36] **Table S2:** Overview of clinical trials which used single dose nevirapine treatment to prevent mother-to-child transmission and which reported the number of patients with nevirapine resistance detected 6 to 8 weeks after treatment. **Table S3:** Overview of clinical trials with structured treatment interruptions which reported the number of patients with at least one drug resistance mutation detected.
(PDF)

Text S3 Computer code, written in C++, which was used to run the simulations, as used for figures 1, 3 and 6.
(TXT)
