# Supplementary material for: Standing Genetic Variation and the Evolution of Drug Resistance in HIV
Source: PLoS Comput Biol. 2012 Jun 7;8(6):e1002527. doi: 10.1371/journal.pcbi.1002527 (PMC3369920; doi:10.1371/journal.pcbi.1002527)
Supplement: Text S1 — Description of the model, the simulations, the calculation of the fixation probability and the data that were used for the analysis. (PDF) [file pcbi.1002527.s001.pdf]

STANDING GENETIC VARIATION AND THE EVOLUTION OF DRUG RESISTANCE IN HIV - SUPPLEMENTARY TEXT S1

PLEUNI S. PENNINGS

Supplementary text S1 for Pennings, P.S., Standing genetic variation and the evolution of drug resistance in HIV, PLoS Comp Biol, 2012

Model. The model describes population dynamics and population genetics of a panmictic virus population in a single patient. Only virus in infected cells is considered and the dynamics of free virus and uninfected cells are ignored. The total number of cells that can be infected is limited to K, the carrying capacity. Because the life cycle of the virus is simplified to just one step in which infected cells "give birth" to infected cells, all drugs have the same effect: they reduce the number of cells that can be infected by the virus in an infected cell. We will refer to the infected cells by the virus they are infected with.

When patients fail therapy, this is often due to virus that is resistant against just one drug even if the patient was treated with three drugs (as described in the Introduction). We therefore assume in our model that there are just two types of viruses: wildtype virus that is susceptible to all drugs and resistant virus that is resistant against one of the drugs in the regimen of the patient. We assume that prior to establishment of single drug-resistant virus (hereafter: resistant virus), there is no virus present that is resistant against more than one drug. We only focus on the probability that single drug-resistant virus becomes established.

The resistant type has a growth rate larger than 1 even in the presence of drugs, but in the absence of drugs it is less fit than the wildtype. Initially the population consists of only wildtype virus, resistant virus can be created by mutation. Absolute fitnesses are determined for wildtype and resistant virus, in the absence of drugs (denoted by u for untreated) and during treatment (either ART or NVP). These fitnesses are absolute fitnesses, which are equivalent to the reproductive ratio, or the expected number of cells a viral particle would infect in a completely susceptible population of host cells. Without drugs, wildtype virus is fitter than resistant virus, so that the population will always be dominated by wildtype virus. During treatment, resistant virus is fitter than wildtype, so that the population will ultimately become dominated by resistant virus. However, if evolution is mutation-limited, it can take a long time before the equilibrium with resistant virus is reached. One of the successes of combination therapy is to make this waiting time much longer than with monotherapy.

The relative cost or selective disadvantage is defined as $C_{rel} = (Fwt_u - Fm_u)/(Fwt_u)$, or, in other words, how much less fit resistant virus is in the absence of drugs, relative to the wildtype. As will be shown later, the fitness of the wildtype with drugs is not important in the current model (although we do have to determine its value for the simulations).

If the average fitness of the viral population is larger than 1, the viral population grows exponentially until it reaches the carrying capacity (K), at which point the population stops growing and stays stable. If the average fitness of the population is below 1, the population shrinks until it reaches $N_{LAT}/(1 - F_{wt})$, where N_{LAT} is a fixed number of particles which is added to the population each generation. This reflects the latently infected cells in an

HIV infected patient. These latently infected cells have very long half-lifes and function as a reservoir for the HIV population. Because of these cells, the virus population does not die out, even if therapy is very successful and there is (almost) no replication.

Simulation details. Time is counted in generations. Each generation is split in 10 time-steps. At each time step, each viral particle has a probability of 1/10 to be chosen to reproduce and die. The expected life-time of a viral particle is therefore 1 generation and this is the same for wildtype and resistant virus. All particles that are chosen to reproduce at a given time-step die and are replaced by their offspring (i.e. newly infected cells). The number of offspring is equal to [number of chosen wildtype particles]$*[F_{wt}]+$[number of chosen resistant particles]$*$ $[F_{res}]$. If this number is smaller than 20, stochastic effects should be taken into account, so instead of using the number directly, we take a random number from the Poisson distribution with the expected number as the mean. If adding of the new offspring to the population would lead the population size to be higher than K, we add fewer individuals so that the population size will be exactly K. The new offspring can be either wildtype or resistant. It is assigned a type at random, with probability

$$\frac{[\text{number of chosen wildtype particles}] * [F_{wt}]}{[\text{number of chosen wildtype particles}] * [F_{wt}] + [\text{number of chosen res particles}] * [F_{res}]}$$

to be wildtype, and resistant otherwise. This is a standard procedure (also used in [1]) The choice of 10 time-steps per generation is a compromise between the speed of simulating discrete generations and the reality of continuous replication.

Mutation occurs only from wildtype to resistant. Back-mutation to wildtype is ignored. The number of mutations in a generation depends on the (per particle and per generation) mutation rate, μ, and the number of newly created infected cells, which in turn depends on fitness and population size.

Analytical calculation of the fixation probability. To calculate the probability that resistance mutations become established when treatment is started (either for the first time or after an interruption), there are two possibilities. When the relative cost of resistance in the absence of drugs, the fitness of the resistant virus with drugs and the viral population size are all known, we can use the theory from [1] to calculate the probability that resistance mutations from the standing genetic variation become established. Alternatively, if the number of copies of the resistant virus and the fitness of resistant virus are known, it is possible to calculate the probability that at least one of these copies will survive, in which case the population becomes resistant. For the latter, we need the probability that a single resistant mutant spreads in the population and ultimately goes to fixation, as opposed to being lost due to genetic drift. In the current model, individuals (viral particles in infected cells) will "produce" a Poisson distributed number of newly infected cells (we call these offspring). The mean number of offspring, λ, determines the probability that a lineage dies out or survives. Usually, it is assumed that the mean number of offspring of a genotype depends on its fitness relative to the mean fitness of the population. However, when a patient takes drugs, the population size of the virus will be much lower than the carrying capacity, so that there is effectively no competition between resistant and wildtype virus. The mean number of offspring, λ, therefore does not depend on the relative fitness, but simply equals the absolute fitness of the resistant virus. In 1927, Haldane [2] showed that the relationship between the mean number of offspring (λ) and the fixation probability is

$$\lambda = -\frac{log(1-P_{fix})}{P_{fix}} = 1 + \frac{P_{fix}}{2} + \frac{P_{fix}^2}{3} + \frac{P_{fix}^3}{4} + \frac{P_{fix}^4}{5} + O(P_{fix}^5) \quad (1)$$

Traditionally, the fixation probability is calculated using the selection coefficient, $s_b = \frac{F_{res}-F_{wt}}{F_{wt}}$, instead of the expected number of offspring, λ. In a population with stable population size and competition between genotypes this is fine because $\lambda = 1 + s_b$, but in the current model, this does not hold and s_b is irrelevant.

If $\lambda - 1$ is small, a simple approximation for the fixation probability is:

$$P_{fix} \approx 2(\lambda - 1) \quad (2)$$

When λ is larger, Haldane's equation needs to be solved numerically using more terms of the Taylor expansion.

Data from clinical trials. Starting standard therapy. The probability that resistance mutations from standing genetic variation become established at the start of therapy was estimated from a data set that was published in [3], see supplementary table S1 in text S2 for the raw data. The study reported the number of patients where resistance was detected in the first, second and third year of treatment, for two treatment groups and for three categories of resistance mutations. Because in each case only the first resistance mutation in a given patient was recorded, the data for the three different resistance classes could not be analyzed separately. A saturated model was fitted using R [4] after which non-significant interactions were removed. It was found that the probability that resistance mutations became established did not differ significantly between the two treatment groups (p=0.26) and also not between year 2 and year 3 (p=0.78). However, there was a clear difference between year 1 and the other two years ($p < 10^{-5}$). The average risk per year in year 2 and 3 was 3.7%, whereas the average risk in year 1 was 9.5%. The difference between year 1 and the other 2 years was 5.8%, which could be due to the standing genetic variation before treatment started.

Single dose nevirapine. We searched the web of science (ISI) database for published studies on single dose nevirapine and resistance. We limited ourselves to studies that reported the fraction of women that had detectable resistance at 4 to 6 weeks postpartum using standard sequencing. An overview of the studies can be found in supplementary table S2 in text S2.

Interruption studies. Seven studies were identified that reported on clinical trials in which patients interrupted treatment multiple times for a fixed length of time (i.e., all patients in the trial were on the same schedule) and which tested genotypic resistance on plasma samples of all patients in which treatment failed (and ideally of all patients) before and after the trial. Characteristics of the seven studies can be found in supplementary table S3 n text S2. The shortest interruption length was two days and the longest 60 days. If possible, we removed patients which had evidence of resistance before the trial started. In all but one of the studies, patients were on traditional triple-drug regimens (NNRTI or PI based with two NRTIs). Only in the Staccato study some patients were on a boosted PI regimen [5]. These patients were taken out of the analysis because resistance seems to evolve much slower in regimens with boosted PI's compared to NNRTI and unboosted PI regimens [6]. In two trials, the interruptions had varying lengths [7, 8], in which case we used the mean length of the interruptions for the plot (see figure 6).

For each study, the fraction of patients (F) which did not acquire any resistance mutations during the trial was calculated. The number of treatment interruptions (T) which were relevant for the resistance data was calculated. For example, if genotypic resistance tests were done on samples that were obtained during treatment interruption 4, it was assumed that any resistance had occurred due to treatment interruption 1, 2 or 3 so that $T = 3$. Furthermore it was assumed that each treatment interruption (TI) contributed equally to the probability that resistance mutations became established. The probability (P) that resistance mutations became established due to a single interruption was calculated as follows:

$$F = (1 - P)^T \Rightarrow P = 1 - F^{1/T} \quad (3)$$

Two of the studies also allowed for an estimation of the risk that resistance mutations became established during continuous treatment [9, 10, 11]. We find a rate of evolution of resistance of 4.3% and 4.8% per year (see table S3 in text S2). Using the mean of these two rates, we can estimate the probability that resistance mutations became established during the time on treatment in each of the trials and we corrected the estimated risk of an interruption by subtracting the probability that resistance mutations became established during treatment.

References

1. Hermisson J, Pennings PS (2005) Soft sweeps: Molecular population genetics of adaptation from standing genetic variation. Genetics 169: 2335-2352.
2. Haldane JBS (1927) A mathematical theory of natural and artificial selection, part v: Selection and mutation. Proceedings of the Cambridge Philosophical Society 23: 838-844.
3. Margot NA, Lu B, Cheng A, Miller MD, Study T (2006) Resistance development over 144 weeks in treatment-naive patients receiving tenofovir disoproxil fumarate or stavudine with lamivudine and efavirenz in study 903. Hiv Medicine 7: 442-450.
4. R Development Core Team (2011) R: A Language and Environment for Statistical Computing. R Foundation for Statistical Computing, Vienna, Austria. URL `http://www.R-project.org`. ISBN 3-900051-07-0.
5. Ananworanich J, Nuesch R, Le Braz M, Chetchotisakd P, Vibhagool A, et al. (2003) Failures of 1 week on, 1 week off antiretroviral therapies in a randomized trial. Aids 17: F33-F37.
6. Lima VD, Gill VS, Yip B, Hogg RS, Montaner JSG, et al. (2008) Increased resilience to the development of drug resistance with modern boosted protease inhibitor-based highly active antiretroviral therapy. Journal of Infectious Diseases 198: 51-58.
7. Hoen B, Fournier I, Lacabaratz C, Burgard M, Charreau I, et al. (2005) Structured treatment interruptions in primary hiv-1 infection - the anrs 100 primstop trial. Jaids-Journal of Acquired Immune Deficiency Syndromes 40: 307-316.
8. Palmisano L, Giuliano M, Bucciardini R, Fragola V, Andreotti M, et al. (2007) Determinants of virologic and immunologic outcomes in chronically hiv-infected subjects undergoing repeated treatment interruptions - the istituto superiore di sanita-pulsed antiretroviral therapy (iss-part) study. Jaids-Journal of Acquired Immune Deficiency Syndromes 46: 39-47.
9. Reynolds SJ, Kityo C, Mbamanya F, Dewar R, Ssali F, et al. (2009) Evolution of drug resistance after virological failure of a first-line highly active antiretroviral therapy regimen in uganda. Antiviral Therapy 14: 293-297.
10. Reynolds SJ, Kityo C, Hallahan CW, Kabuye G, Atwiine D, et al. (2010) A randomized, controlled, trial of short cycle intermittent compared to continuous antiretroviral therapy for the treatment of hiv infection in uganda. Plos One 5.
11. Danel C, Moh R, Chaix ML, Gabillard D, Gnokoro J, et al. (2009) Two-months-off, four-months-on antiretroviral regimen increases the risk of resistance, compared with continuous therapy: A randomized trial involving west african adults. Journal of Infectious Diseases 199: 66-76.
