# Supplementary material for: Standing Genetic Variation and the Evolution of Drug Resistance in HIV
Source: PLoS Comput Biol. 2012 Jun 7;8(6):e1002527. doi: 10.1371/journal.pcbi.1002527 (PMC3369920; doi:10.1371/journal.pcbi.1002527)
Supplement: Text S2 — includes tables S1, S2, S3. Table S1: Number of patients with at least one resistance mutation detected by the end of the first, second and third year of NNRTI-based antiretroviral therapy. Data from [36] Table S2: Overview of clinical trials which used single dose nevirapine treatment to prevent mother-to-child transmission and which reported the number of patients with nevirapine resistance detected 6 to 8 weeks after treatment. Table S3: Overview of clinical trials with structured treatment interruptions which reported the number of patients with at least one drug resistance mutation detected. (PDF) [file pcbi.1002527.s002.pdf]

# STANDING GENETIC VARIATION AND THE EVOLUTION OF DRUG RESISTANCE IN HIV - TEXT S2 WITH TABLES S1, S2, S3

PLEUNI S. PENNINGS

Supplementary tables S1, S2, S3 for Pennings, P.S., Standing genetic variation and the evolution of drug resistance in HIV, PLoS Comp Biol, 2012

**Table S1.** Number of patients with at least one resistance mutation detected by the end of the first, second and third year of NNRTI-based antiretroviral therapy. Data from [1].

| Year | Group | Resistant | Not Resistant |
|---|---|---|---|
| 1 | TDF | 35 | 264 |
| 2 | TDF | 8 | 256 |
| 3 | TDF | 9 | 247 |
| 1 | d4T | 22 | 279 |
| 2 | d4T | 10 | 269 |
| 3 | d4T | 11 | 258 |

**Table S2.** Overview of clinical trials with single dose nevirapine which reported the number of patients with nevirapine resistance detected 6 to 8 weeks after treatment.

| Reference and name trial | Number of patients | Treatment | Treatment code | Est. prob of establishment of resistance | Remarks |
|---|---|---|---|---|---|
| Eshleman 2005 [2], Malawi | 65 | sdNVP | sdNVP | 0.69 | |
| Eshleman 2005 [2], Uganda | 241 | sdNVP | sdNVP | 0.26 | |
| Farr 2010 [3], Malawi | 65 | sdNVP | sdNVP | 0.34 | |
| Hudelson 2010 [4], Uganda | 30 | sdNVP | sdNVP | 0.43 | |
| Jackson 2000 [5], Uganda | 15 | sdNVP | sdNVP | 0.20 | |
| Kassaye 2007 [6], Zimbabwe | 32 | sdNVP | sdNVP | 0.35 | |
| Lee 2005 [7], Zimbabwe | 32 | sdNVP | sdNVP | 0.34 | |
| Loubser 2006 [8], South Africa | 44 | sdNVP | sdNVP | 0.54 | Results only for K103N mutation |
| Ly 2007 [9], Cambodia | 35 | sdNVP | sdNVP | 0.23 | |
| Martinson 2009 [10], South Africa, HIVNET 012 | 108 | sdNVP | sdNVP | 0.38 | |
| Martinson 2009 [10], South Africa, HIVNET 012 | 193 | sdNVP | sdNVP | 0.46 | |
| McIntyre 2009 [11], South Africa | 74 | sdNVP | sdNVP | 0.59 | |
| Rajesh 2010 [12], India | 12 | sdNVP | sdNVP | 0.33 | |
| Toni 2005 [13] , Ivorycoast | 29 | sdNVP | sdNVP | 0.21 | |
| Farr 2010 [3], Malawi | 120 | sdNVP + 7 days 3TC/ZDV | sdNVP/PP | 0.017 | |
| McIntyre 2009 [11], South Africa | 164 | sdNVP + 4 days 3TC/ZDV | sdNVP/PP | 0.097 | |
| McIntyre 2009 [11], South Africa | 168 | sdNVP + 7 days 3TC/ZDV | sdNVP/PP | 0.073 | |
| Chaix 2007 [14], Ivorycoast, ANRS/Ditrame Plus | 63 | ZDV from 36 weeks + sdNVP | ZDV/sdNVP | 0.33 | |
| Chalermchokcharoenkit 2009 [15], Thailand | 190 | ZDV 3rd trimester + sdNVP | ZDV/sdNVP | 0.18 | |
| Chi 2009 and 2007 [16, 17], Zambia | 166 | ZDV 3rd trimester + sdNVP | ZDV/sdNVP | 0.25 | |
| Lallemant 2009 [18], Thailand, PHPT2 | 222 | ZDV 3rd trimester + sdNVP | ZDV/sdNVP | 0.064 | |
| Ly 2007 [9], Cambodia | 16 | ZDV from 28 weeks + sdNVP | ZDV/sdNVP | 0.19 | |
| Shapiro 2006 [19], Botswana | 155 | ZDV from 34 weeks + sdNVP | ZDV/sdNVP | 0.45 | |
| Van Zijl 2008 [20], South Africa | 76 | ZDV from 34 weeks + sdNVP | ZDV/sdNVP | 0.17 | |
| Arrive 2010 [21], Cambodia/Ivorycoast/South Africa, TEmAA ANRS 12109 | 33 | ZDV from enrollment + sdNVP + 1 week TDF/FTC | ZDV/sdNVP/PP | 0.0 | |
| Dabis 2009 [22], Cambodia/Ivorycoast/South Africa, TEmAA ANRS 12109 | 37 | ZDV from enrollment + sdNVP + 1 week TDF/FTC | ZDV/sdNVP/PP | 0.0 | |
| Lallemant 2009 [18], Thailand, PHPT4 | 222 | ZDV 3rd trim + sdNVP + 1 month ZDV/DDI | ZDV/sdNVP/PP | 0.0 | |

**Table S3.** Overview of clinical trials with structured treatment interruptions which reported the number of patients with at least one drug resistance mutation detected.

| Reference and name trial | % on PI | Number of patients | Patients excluded | Patients with genotypic resistance | Fraction not resistant at end of trial | TI's relevant for calculation | Length of TI (days) | Treatment period (days) | Est. prob of evolution of resistance per TI | Corrected prob of evolution of resistance |
|---|---|---|---|---|---|---|---|---|---|---|
| Interruption arms | | | | | | | | | | |
| Reynolds 2009 and 2010 [23, 24], Uganda FOTO arm | 2% | 57 | 1 (no genotype) | 4 | 52/56 | 72 | 2 | 5 | 0 | 0 |
| Ananworanich 2003 [25], Staccato WOWO arm | 0% | 36 | 22 (on boosted PI) 2 (had resistance before trial) | 3 | 9/12 | 17 | 7 | 7 | 0.02 | 0.02 |
| Reynolds 2009 and 2010 [23, 24], Uganda WOWO arm | 6% | 32 | 3 (left the trial) | 9 | 20/29 | 18 | 7 | 7 | 0.02 | 0.02 |
| Yerly 2003 [26] , SSITT trial | 100% | 87 | 4 (lost to follow-up) 3 (already resistance before trial) | 11 | 69/80 | 4 | 14 | 56 | 0.04 | 0.03 |
| Hoen 2005 [27], ANRS 100 Primstop trial | 100% | 26 | 4 (resistance before trial) 1 (resistance in first TI) | 2 | 19/21 | 2 | 14, 28 (mean 21) | 84 | 0.05 | 0.04 |
| Palmisano 2007 [28], ISS_PART | 25% | 136 | 16 (estimated number that had resistance before) | 22 | 98/120 | 3 | 28, 28, 56 (mean 37) | 91 | 0.07 | 0.06 |
| Danel 2009 [29], Ivory Coast NCT 00158405 | 10% | 325 | 10 (no genotypes) | 76 | 239/315 | 4 | 60 | 91 | 0.07 | 0.06 |
| Continuous arms | | | | | | | | | | |
| Reynolds 2009 and 2010 [23, 24], Uganda continuous arm | 2% | 51 | 0 | 3 | 48/51 | – | – | 504 | – | 0.043/year |
| Danel 2009 [29], Ivory Coast NCT 00158405 | 15% | 110 | 3 (no genotypes) | 10 | 97/107 | – | – | 728 | – | 0.048/year |

# References


1. Margot NA, Lu B, Cheng A, Miller MD, Study T (2006) Resistance development over 144 weeks in treatment-naive patients receiving tenofovir disoproxil fumarate or stavudine with lamivudine and efavirenz in study 903. Hiv Medicine 7: 442-450.
2. Eshleman SH, Guay LA, Wang J, Mwatha A, Brown ER, et al. (2005) Distinct patterns of emergence and fading of k103n and y181c in women with subtype a vs. d after single-dose nevirapine - hivnet 012. Jaids-Journal of Acquired Immune Deficiency Syndromes 40: 24-29.

3. Farr SL, Nelson JAE, Ng'ombe TJ, Kourtis AP, Chasela C, et al. (2010) Addition of 7 days of zidovudine plus lamivudine to peripartum single-dose nevirapine effectively reduces nevirapine resistance postpartum in hiv-infected mothers in malawi. Jaids-Journal of Acquired Immune Deficiency Syndromes 54: 515-523.
4. Hudelson SE, McConnell MS, Bagenda D, Piwowar-Manning E, Parsons TL, et al. (2010) Emergence and persistence of nevirapine resistance in breast milk after single-dose nevirapine administration. Aids 24: 557-561.
5. Jackson JB, Becker-Pergola G, Guay LA, Musoke P, Mracna M, et al. (2000) Identification of the k103n resistance mutation in ugandan women receiving nevirapine to prevent hiv-1 vertical transmission. Aids 14: F111-F115.
6. Kassaye S, Lee E, Kantor R, Johnston E, Winters M, et al. (2007) Drug resistance in plasma and breast milk after single-dose nevirapine in subtype c hiv type 1: Population and clonal sequence analysis. Aids Research and Human Retroviruses 23: 1055-1061.
7. Lee EJ, Kantor R, Zijenah L, Sheldon W, Emel L, et al. (2005) Breast-milk shedding of drug-resistant hiv-1 subtype c in women exposed to single-dose nevirapine. Journal of Infectious Diseases 192: 1260-1264.
8. Loubser S, Balfe P, Sherman G, Hammer S, Kuhn L, et al. (2006) Decay of k103n mutants in cellular dna and plasma rna after single-dose nevirapine to reduce mother-to-child hiv transmission. Aids 20: 995-1002.
9. Ly N, Phoung V, Min DC, Srey C, Kruy LS, et al. (2007) Reverse transcriptase mutations in cambodian crf01ae isolates after antiretroviral prophylaxis against hiv type 1 perinatal transmission. Aids Research and Human Retroviruses 23: 1563-1567.
10. Martinson NA, Morris L, Johnson J, Gray GE, Pillay V, et al. (2009) Women exposed to single-dose nevirapine in successive pregnancies: effectiveness and nonnucleoside reverse transcriptase inhibitor resistance. Aids 23: 809-816.
11. McIntyre JA, Hopley M, Moodley D, Eklund M, Gray GE, et al. (2009) Efficacy of short-course azt plus 3tc to reduce nevirapine resistance in the prevention of mother-to-child hiv transmission: A randomized clinical trial. Plos Medicine 6: 9.
12. Rajesh L, Ramesh K, Hanna LE, Narayanan PR, Swaminathan S (2010) Emergence of drug resistant mutations after single dose nevirapine exposure in hiv-1 infected pregnant women in south india. Indian Journal of Medical Research 132: 509-512.
13. Toni TD, Masquelier B, Lazaro E, Dore-Mbami M, Ba-Gomis FO, et al. (2005) Characterization of nevirapine (nvp) resistance mutations and hiv type 1 subtype in women from abidjan (cote d'ivoire) after nvp single-dose prophylaxis of hiv type 1 mother-to-child transmission. Aids Research and Human Retroviruses 21: 1031-1034.
14. Chaix ML, Ekouevi DK, Peytavin G, Rouet F, Tonwe-Gold B, et al. (2007) Impact of nevirapine (nvp) plasma concentration on selection of resistant virus in mothers who received single-dose nvp to prevent perinatal human immunodeficiency virus type 1 transmission and persistence of resistant virus in their infected children. Antimicrobial Agents and Chemotherapy 51: 896-901.
15. Chalermchockcharoenkit A, Culnane M, Chotpitayasunondh T, Vanprapa N, Leelawiwat W, et al. (2009) Antiretroviral resistance patterns and hiv-1 subtype in mother-infant pairs after the administration of combination short-course zidovudine plus single-dose nevirapine for the prevention of mother-to-child transmission of hiv. Clinical Infectious Diseases 49: 299-305.
16. Chi BH, Ellis GM, Chintu N, Cantrell RA, Sinkala M, et al. (2009) Intrapartum tenofovir and emtricitabine reduces low-concentration drug resistance selected by single-dose nevirapine for perinatal hiv prevention. Aids Research and Human Retroviruses 25: 1099-1106.
17. Chi BH, Sinkala M, Mbewe F, Cantrell RA, Kruse G, et al. (2007) Single-dose tenofovir and emtricitabine for reduction of viral resistance to non-nucleoside reverse transcriptase inhibitor drugs in women given intrapartum nevirapine for perinatal hiv prevention: an open-label randomised trial. Lancet 370: 1698-1705.
18. Lallemant M, Ngo-Giang-Huong N, Jourdain G, Traisaithit P, Cressey TR, et al. (2010) Efficacy and safety of 1-month postpartum zidovudine-didanosine to prevent hiv-resistance mutations after intrapartum single-dose nevirapine. Clinical Infectious Diseases 50: 898-908.

19. Shapiro RL, Thior I, Gilbert PB, Lockman S, Wester C, et al. (2006) Maternal single-dose nevirapine versus placebo as part of an antiretroviral strategy to prevent mother-to-child hiv transmission in botswana. Aids 20: 1281-1288.
20. van Zyl GU, Claassen M, Engelbrecht S, Laten JD, Cotton MF, et al. (2008) Zidovudine with nevirapine for the prevention of hiv mother-to-child transmission reduces nevirapine resistance in mothers from the western cape, south africa. Journal of Medical Virology 80: 942-946.
21. Arrive E, Chaix ML, Nerrienet E, Blanche S, Rouzioux C, et al. (2010) Maternal and nenonatal tenofovir and emtricitabine to prevent vertical transmission of hiv-1: tolerance and resistance. Aids 24: 2478-2485.
22. Dabis F, Grp TEAS (2009) Tolerance and viral resistance after single-dose nevirapine with tenofovir and emtricitabine to prevent vertical transmission of hiv-1. Aids 23: 825-833.
23. Reynolds SJ, Kityo C, Hallahan CW, Kabuye G, Atwiine D, et al. (2010) A randomized, controlled, trial of short cycle intermittent compared to continuous antiretroviral therapy for the treatment of hiv infection in uganda. Plos One 5.
24. Reynolds SJ, Kityo C, Mbamanya F, Dewar R, Ssali F, et al. (2009) Evolution of drug resistance after virological failure of a first-line highly active antiretroviral therapy regimen in uganda. Antiviral Therapy 14: 293-297.
25. Ananworanich J, Nuesch R, Le Braz M, Chetchotisakd P, Vibhagool A, et al. (2003) Failures of 1 week on, 1 week off antiretroviral therapies in a randomized trial. Aids 17: F33-F37.
26. Yerly S, Fagard C, Gunthard HF, Hirschel B, Perrin L, et al. (2003) Drug resistance mutations during structured treatment interruptions. Antiviral Therapy 8: 411-415.
27. Hoen B, Fournier I, Lacabaratz C, Burgard M, Charreau I, et al. (2005) Structured treatment interruptions in primary hiv-1 infection - the anrs 100 primstop trial. Jaids-Journal of Acquired Immune Deficiency Syndromes 40: 307-316.
28. Palmisano L, Giuliano M, Bucciardini R, Fragola V, Andreotti M, et al. (2007) Determinants of virologic and immunologic outcomes in chronically hiv-infected subjects undergoing repeated treatment interruptions - the istituto superiore di sanita-pulsed antiretroviral therapy (iss-part) study. Jaids-Journal of Acquired Immune Deficiency Syndromes 46: 39-47.
29. Danel C, Moh R, Chaix ML, Gabillard D, Gnokoro J, et al. (2009) Two-months-off, four-months-on antiretroviral regimen increases the risk of resistance, compared with continuous therapy: A randomized trial involving west african adults. Journal of Infectious Diseases 199: 66-76.
